# Adverse childhood experiences among patients with substance use disorders at a referral psychiatric hospital in Kenya

**DOI:** 10.1186/s12888-018-1780-1

**Published:** 2018-06-18

**Authors:** Sarah Kanana Kiburi, Keneilwe Molebatsi, Anne Obondo, Mary W. Kuria

**Affiliations:** 1 Department of Psychiatry, Mbagathi Hospital, Nairobi County, Nairobi, P.O. BOX 20725-00202 Kenya; 2 Department of Psychiatry, University of Botswana, Private Bag, 00713 Gaborone, Botswana; 3 Department of Psychiatry, University of Nairobi, Nairobi, P.O. BOX 19676-00202 Kenya; 4 Department of Psychiatry, University of Nairobi, Nairobi, P.O. BOX 19676-00202 Kenya

**Keywords:** Adverse childhood experiences, Substance use disorders, Patients, Kenya

## Abstract

**Background:**

Substance use disorders are a major cause of health and social problems worldwide. Research evidence shows a strong graded relationship of adverse childhood experiences and substance use in adulthood. This study aimed at determining the prevalence of adverse childhood experiences and their association with substance use among patients with substance use disorders.

**Method:**

The study used a descriptive cross-sectional design. A total of 134 patients aged 18 years and above receiving inpatient treatment for substance use disorders were recruited into the study. A mental state exam was done to rule out active psychopathology. Data on socio demographic variables, adverse childhood experiences (ACEs) and substance use was collected using Adverse Childhood Experiences International Questionnaire and The Alcohol, Smoking and Substance Involvement Screening Test respectively. Data was analysed using statistical package for social sciences (SPSS) version 20 for windows.

**Results:**

Males accounted for the majority of the study participants (*n* = 118, 88.1%). Only 43.3% (*n* = 58) of the participants had a family history of substance use disorder. The most frequently used substance was alcohol which was reported by 82.1% of the participants. Nearly 93% of the respondents had experienced at least one ACE and the most prevalent ACE was one or no parent which was reported by half of the respondents. The adverse childhood experiences significantly associated with current problematic substance use were; emotional abuse, having someone with mental illness in the household, physical abuse and physical neglect. Emotional abuse significantly predicted tobacco (A.O.*R* = 5.3 (1.2–23.9)) and sedative (A.O.*R* = 4.1 (1.2–14.2)) use. Childhood exposure to physical abuse was associated with cannabis use [A.O.*R* = 2.9 (1.0–7.9)]. Experiencing five or more ACEs was associated with increased risk of using sedatives.

**Conclusion:**

There is a high prevalence of adverse childhood experiences among patients with substance use disorders. Experiencing emotional abuse, having someone with mental illness in the household, physical abuse and physical neglect in childhood are risk factors of substance use disorders. ACEs screening and management should be incorporated in substance abuse prevention programs and policies.

## Background

Childhood experiences have a tremendous impact on the development and prevalence of a wide range of health problems throughout a person’s lifespan [1]. Early research conducted in this subject has been referred to as Adverse Childhood Experiences [2]. Adverse childhood experiences (ACEs) refer to stressful or traumatic events that children may suffer during early life [3]. Examples of such experiences include: various types of abuse, dysfunctional households, neglect and living in a violent community [4]. ACEs have been linked to substance use and behavioural disorders [1, 5]; as the number of ACEs increases, so does the risk for these outcomes.

Exposure to repeated stress in children may result in disruption of their neurodevelopment [1, 6]. This will result in impaired cognitive functioning. Over time, especially during adolescence the affected child tends to resort to negative coping mechanisms such as self-harm or substance use to cope with stressful life events [7, 8].

Substance use problems are prevalent and widespread worldwide. The World Health Organization (WHO) identified alcohol, tobacco and illicit drug use as among the top 20 risk factors for ill health. Globally 12.6% of deaths are attributable to alcohol, tobacco and illicit drug use. Tobacco alone causes almost one in eight deaths among adults aged 30 years and above. Substance use disorders account for 9 % of disability-adjusted life year (DALYs) worldwide [3]. A clinical epidemiology study conducted among patients admitted at a psychiatric hospital in Kenya reported that 34.4% of admitted patients met the criteria for substance use disorders [9]. Another study conducted in rural and urban health centres in Kenya found that alcohol, tobacco, khat and cannabis were the commonest substances used in Kenya [10].

Research has demonstrated a strong positive association between ACEs and substance use disorders [11–14]. There is also evidence that presence of ACEs has a negative influence on management of patients with substance use disorders [15–17]. This indicates the need for screening of ACEs so that prevention measures could be put in place after identification of those at risk.

Most studies done on ACEs and their association with substance use are in developed countries [18, 19] with few studies done in developing countries on ACEs and their influence on various health outcomes. These include a study conducted in Nigeria on ACEs as risk factors for psychiatric illness including substance abuse. Another study conducted in four sub-Saharan African countries (Ghana, Burkina Faso, Uganda and Malawi) found an association between experience of ACEs and drunkenness in adolescence [14]. Most of the studies that have been done in Kenya on substance abuse focus on the prevalence of substance abuse without any association with risk factors. There is only one recently published study which studied ACE association with alcohol consumption patterns among Kenyan mothers [20]. This creates a knowledge gap that needs attention. This study therefore aimed at establishing the prevalence of ACEs and their association with substance use among patients with substance use disorders. The data obtained will inform policy makers, and clinicians on ACEs and their association with substance use disorders. This will help in selection and implementation of policies, programs and strategies designed to address ACEs and include them in substance use prevention and treatment.

## Methods

### Study design and duration

This was a descriptive cross-sectional study. Data collection was done over a period of 2 months.

### Study setting and population

This study was conducted at Mathari National Teaching and Referral Hospital (MNTRH) in Nairobi which is the only referral psychiatric hospital in Kenya. The study targeted inpatients with substance use disorders. To participate in the study patients had to be: aged 18 years and above, mentally stable at the time of the interview and able to give informed consent. The participants were drawn from the inpatient wards (male and female) and the drug rehabilitation unit. In the general wards patients on management for substance use disorders were identified from the admission record book in the ward.

### Procedure

Study participants were recruited using convenience sampling method. All patients on management for substance use disorders were approached, screened for inclusion criteria and invited to participate until the desired sample size (134) was met. The researcher conducted a mental state exam to rule out active psychopathology (those with delusions, hallucinations and lacking insight). Those found to have active psychopathology were excluded.

Those who met the inclusion criteria and were willing to participate, were given a consent document to read and then after addressing their questions or concerns they were asked to sign an informed consent form. Data collection then proceeded by utilising ACE-IQ and ASSIST questionnaire for socio demographic data, adverse childhood experiences and substance use.

### Measures and instruments

#### Adverse childhood experiences international questionnaire, (ACE- IQ)

The questionnaire was designed by WHO and Centres for Disease Control and Preventions (CDC) to measure ACEs in all countries and association between them and risk behaviours later in life [4]. The tool is currently being validated in several countries through trial implementation as part of broader health survey [21]. The ACE-IQ is designed for administering to people aged 18 years and above.

The questionnaire has a section that captures data on the socio-demographic variables of study participants, which include: age, sex, education level, ethnic group, marital status and employment. The researchers modified this section to leave out the section that asks about ethnic group and included a question about age at which participants first used substances of abuse. The ACEs screened by the questionnaire are: physical abuse, emotional abuse, sexual abuse, physical neglect, emotional neglect, household member treated violently, household member who was a substance abuser, household member with mental illness or suicidal, incarcerated household member, peer violence (bullying), community violence and collective violence. Two methods of analysis of ACE-IQ have been proposed [22]. For this study the frequency version of analysis was used. Total score was calculated by summing up the number of events a participant was exposed to.

ACE IQ has been found to be a reliable and valid index of the adverse childhood experience in Africa [23]. Another study in Germany described the ACE-IQ as a reliable, valid and economical screening tool for adverse childhood experiences [24].

#### Alcohol, smoking and substance involvement screening test (ASSIST)

The tool is used to screen for problematic or risky substance use. ASSIST consists of sections that have questions on tobacco, alcohol, cannabis, cocaine, amphetamine, inhalants, sedatives, hallucinogen, opioids and other drugs [25].

In validation studies done, ASSIST has shown good concurrent validity (*r* = 0.76–0.88, *p* ≤ 0.001), good internal consistency (α = 0.68–0.88) and ability to discriminate between gradation of abuse [26–28]. A validation study done on adolescents reported high validity with sensitivities of 95–100%, specificities of 90–93% and area under the curve, AUC, 90–94% [26].

#### Data analysis

Descriptive statistics was conducted to summarize socio-demographic characteristics, family history of mental illness and substance use. Chi-square/Fischer’s exact tests was used to test for the heterogeneity between males and females. Prevalence rates and their 95% Confidence interval was reported for each of the category of adverse childhood experience (ACEs); Alcohol and substance use (lifetime and current). Prevalence rates were used to describe exposure to different adverse childhood experiences among patients with substance use disorders. Adjusted odds ratio and 95% confidence interval were obtained from logistic regression models that assessed the associations between each category of (ACE) and the common drugs and substances used. The number of ACEs, were summed for each person using Frequency version of analysis guidelines for analysing the ACE-IQ which ranged from 0 to 13; Analysis were repeated with the summed scores as ordinal variable (0, 1, 2, 3, 4, 5 and ≥ 6) or a dichotomous variable (Yes/No) with 0 experience as referent. Covariates in the models included; gender, educational level, employment status, marital status, family history of substance abuse and family history of mental illness.

## Results

The socio-demographic characteristics of the participants are presented in Table 1. Majority of the participants were males (88.1%). Most of the participants were aged between 31 and 40 years (33.6%) and most reported to have first used drugs when they were between the ages of 16 and 20. Family history of substance abuse was reported by 43.3% of the participants and 15.7% reported family history of mental illness.

**Table 1:** Socio-Demographic Characteristics by Gender

Variable	Category	Overall (*N* = 134)	Females (*n* = 16)	Males (*n* = 118)	*P*-Value^a^
		n (%)	n (%)	n (%)	
Gender	Female	16 (11.9)	–	–	–
	Male	118 (88.1)	–	–	
Age in years	18-20 Years	12 (9.0)	1 (6.3)	11 (9.3)	0.859
	21-25 Years	34 (25.4)	4 (25.0)	30 (25.4)	
	26-30 Years	26 (19.4)	3 (18.8)	23 (19.5)	
	31-40 Years	45 (33.6)	7 (43.8)	38 (32.2)	
	Above 40 Years	17 (12.7)	1 (6.3)	16 (13.6)	
Age at first use	15 Years and below	33 (24.6)	2 (12.5)	31 (26.3)	0.455
	16–20 Years	67 (50.0)	10 (62.5)	57 (48.3)	
	21 years and above	34 (25.4)	4 (25.0)	30 (25.4)	
Education level	Primary and below	35 (26.1)	10 (62.5)	25 (21.2)	0.002
	Secondary education	41 (30.6)	3 (18.8)	38 (32.2)	
	Tertiary and above	58 (43.3)	3 (18.8)	55 (46.6)	
Employment status	Unemployed	31 (23.1)	3 (18.8)	28 (23.7)	0.763
	Employed	103 (76.9)	13 (81.3)	90 (76.3)	
Monthly net Income (*N* = 103)	Less than 20,000	49 (47.6)	8 (61.5)	41 (45.6)	0.485
	20,001–35,000	37 (35.9)	4 (30.8)	33 (36.7)	
	Above 35,000	17 (16.5)	1 (7.7)	16 (17.8)	
Marital Status	Married	44 (32.8)	3 (18.8)	41 (34.7)	0.466
	Divorced/ Separated/widowed	25 (18.7)	3 (18.8)	22 (18.6)	
	Single	65 (48.5)	10 (62.5)	55 (46.6)	
Family history of substance use	No	76 (56.7)	10 (62.5)	66 (55.9)	0.789
	Yes	58 (43.3)	6 (37.5)	52 (44.1)	
Family history of mental illness	No	113 (84.3)	15 (93.8)	98 (83.1)	0.320
	Yes	21 (15.7)	1 (6.3)	20 (16.9)	

^a^Chi-square/Fischer’s exact test for heterogeneity test

As depicted in Table 2 the most commonly reported ACE was one or no parent followed by household member treated violently. Males reported experiencing more ACEs compared to females.

**Table 2:** Prevalence and 95% C.I of Categories of ACE and ACE Score by Gender

Category of ACE	Overall(*N* = 134)		Females(*n* = 16)		Males(*n* = 118)	
	n	Prevalence (95% C.I)	n	Prevalence (95% C.I)	n	Prevalence (95% C.I)
Abuse						
Physical abuse	31	23.1 (16.4–29.9)	3	18.8 (0.0–37.5)	28	23.7 (16.1–31.4)
Emotional abuse	30	22.4 (15.7–29.9)	3	18.8 (0.0–37.5)	27	22.9 (15.3–30.5)
Contact sexual	35	26.1 (19.4–33.6)	8	50.0 (25.0–75.0)	27	22.9 (15.3–29.7)
Family						
Alcohol/drug user in household	58	43.3 (35.1–51.5)	6	37.5 (18.8–62.5)	52	44.1 (34.7–52.5)
Incarcerated household member	30	22.4 (15.7–29.9)	4	25.0 (6.3–50.0)	26	22.0 (15.3–29.7)
Someone with mental illness in household	21	15.7 (9.7–22.4)	1	6.3 (0.0–18.8)	20	16.9 (11.0–23.7)
Household member treated violently	66	49.3 (40.3–58.2)	7	43.8 (18.8–68.8)	59	50.0 (41.5–59.3)
One or no parent	67	50.0 (41.0–58.2)	12	75.0 (50.0–93.8)	55	46.6 (37.3–55.9)
Parents/guardian						
Emotional neglect	53	39.6 (30.6–48.5)	6	37.5 (12.5–62.5)	47	39.8 (30.5–48.3)
Physical neglect	16	11.9 (6.7–17.2)	2	12.5 (0.0–31.3)	14	11.9 (6.8–17.8)
Bullying	17	12.7 (7.5–18.7)	0	0.0 (0.0–0.0)	17	14.4 (8.5–20.3)
Violence						
Community violence	41	30.6 (23.1–38.8)	4	25.0 (6.3–50.0)	37	31.4 (22.9–40.7)
Collective violence	55	41.0 (32.8–49.3)	4	25.0 (6.3–43.8)	51	43.2 (33.9–51.7)
ACE Score						
0	10	7.5 (3.7–11.9)	2	12.5 (0.0–31.3)	8	6.8 (2.5–11.9)
1	19	14.2 (9.0–20.1)	0	0.0 (0.0–0.0)	19	16.1 (9.3–22.9)
2	21	15.7 (9.7–21.6)	2	12.5 (0.0–31.3)	19	16.1 (10.2–22.9)
3	17	12.7 (7.5–17.9)	2	12.5 (0.0–31.3)	15	12.7 (6.8–19.5)
4	18	13.4 (8.2–19.4)	4	25.0 (6.3–50.0)	14	11.9 (6.8–17.8)
5	14	10.4 (5.2–15.7)	4	25.0 (6.3–50.0)	10	8.5 (3.4–13.6)
> =6	35	26.1 (18.7–33.6)	2	12.5 (0.0–31.3)	33	28.0 (20.3–36.4)

Table 3 presents the results of the interrelatedness of ACEs, if a respondent was exposed to one of ACEs the probability of exposure to any additional category increased substantially. The median probability of exposure to any additional category given exposure to the first was 97%; for any two additional categories, the median probability was 89%.

**Table 3:** Prevalence of Reporting Of Additional Categories of Aces among Respondents Who Reported Exposure to the First Category

First category of ACE		Physical Abuse (ACE1)	Emotional abuse (ACE2)	Contact sexual abuse (ACE3)	Alcohol/ drug user in household (ACE4)	Incarcerated Household member (ACE5)	Mental illness in the household (ACE6)	household Member treated violently (ACE7)	One or no parent (ACE8)	Emotional neglect (ACE9)	Physical neglect (ACE10)	Bullying (ACE11)	Community violence (ACE12)	Collective violence (ACE13)	Additional ACEs	
															ACE> = 1	ACE> = 2
	N															
ACE1	31	–	65	45	48	29	23	68	58	58	23	10	48	55	100	94
ACE2	30	67	–	47	53	43	33	73	70	57	23	17	53	57	100	93
ACE3	35	40	40	–	54	37	29	57	54	54	20	11	49	46	97	89
ACE4	58	26	28	33	–	24	22	62	57	34	16	10	38	50	95	79
ACE5	30	30	43	43	47	–	47	63	70	67	23	13	47	53	97	97
ACE6	21	33	48	48	62	67	–	62	52	62	29	14	52	57	95	90
ACE7	66	32	33	30	55	29	20	–	67	50	18	18	42	56	95	89
ACE8	67	27	31	28	49	31	16	66	–	46	15	12	37	49	91	84
ACE9	53	34	32	36	38	38	25	62	58	–	9	15	36	58	96	87
ACE10	16	44	44	44	56	44	38	75	63	31	–	25	63	44	94	88
ACE11	17	18	29	24	35	24	18	71	47	47	24	–	59	41	100	88
ACE12	41	37	39	41	54	34	27	68	61	46	24	24	–	59	100	93
ACE13	55	31	31	29	53	29	22	67	60	56	13	13	44	–	98	89
Median		33	36	39	53	33	24	66	59	52	21	14	48	54	97	89
Range		18–67	28–65	24–48	35–62	24–67	16–47	57–75	47–70	31–67	9–29	10–25	36–63	41–59	91–100	79–97

Table 4 presents the results of prevalence of alcohol, tobacco and illicit drugs use in their lifetime and the current use. The most common substance used was alcohol which was being currently used by 82.1% of the participants. Less than a tenth (9.7%) of the participants reported use of only a single substance with majority reporting use of at least 2 substances. As can be seen, the larger population of 26.9% had used 4 different substances within the past 3 months with 14.2% currently using more than 5 substances.

**Table 4:** Prevalence of drug use and number of drugs used by gender

Type of Drug	Overall (*N* = 134)		Females (*n* = 16)		Males (*n* = 118)	
	n	Prevalence (95% C.I)	n	Prevalence (95% C.I)	n	Prevalence (95% C.I)
Lifetime use (Ever used)						
Tobacco	113	84.3 (78.4–90.3)	14	87.5 (68.8–100.0)	99	83.9 (77.1–90.7)
Alcohol	123	91.8 (87.3–96.3)	14	87.5 (68.8–100.0)	109	92.4 (87.3–97.4)
Cannabis	86	64.2 (56.0–72.4)	13	81.3 (62.5–100.0)	73	61.9 (53.4–70.3)
Cocaine	7	5.2 (2.2–9.0)	2	12.5 (0.0–31.3)	5	4.2 (0.8–8.5)
Amphetamine	4	3.0 (0.7–6.0)	0	0.0 (0.0–0.0)	4	3.4 (0.8–6.8)
Inhalants	7	5.2 (1.5–9.0)	0	0.0 (0.0–0.0)	7	5.9 (1.7–10.2)
Sedatives	30	22.4 (15.7–29.1)	4	25.0 (6.3–50.0)	26	22.0 (15.3–30.5)
Hallucinogen	5	3.7 (0.7–7.4)	1	6.3 (0.0–18.8)	4	3.4 (0.8–6.8)
Opioids	11	8.2 (3.7–13.4)	5	31.3 (12.5–56.3)	6	5.1 (1.7–9.3)
Khat	74	55.2 (47.0–64.2)	11	68.8 (43.8–93.8)	63	53.4 (44.9–61.9)
Current use (Past 3 months)						
Tobacco	100	74.6 (67.2–82.1)	13	81.3 (62.5–100.0)	87	73.7 (66.1–81.4)
Alcohol	110	82.1 (75.4–88.8)	13	81.3 (62.5–100.0)	97	82.2 (74.6–89.0)
Cannabis	76	56.7 (49.3–64.9)	11	68.8 (43.8–93.6)	65	55.1 (46.6–64.4)
Cocaine	5	3.7 (0.7–7.5)	0	0.0 (0.0–0.0)	5	4.2 (0.8–8.5)
Amphetamine	3	2.2 (0.0–5.2)	0	0.0 (0.0–0.0)	3	2.5 (0.0–5.9)
Inhalants	5	3.7 (0.7–7.4)	0	0.0 (0.0–0.0)	5	4.2 (0.8–7.6)
Sedatives	19	14.2 (8.2–20.1)	1	6.3 (0.0–18.8)	18	15.3 (9.3–22.0)
Hallucinogen	3	2.2 (0.0–4.5)	0	0.0 (0.0–0.0)	3	2.5 (0.0–5.9)
Opioids	6	4.5 (1.5–8.2)	3	18.8 (0.0–37.5)	3	2.5 (0.0–5.9)
Khat	62	46.3 (38.1–55.2)	9	56.3 (31.3–81.3)	53	44.9 (36.4–53.4)
Number of drugs currently using						
1.00	13	9.7 (5.2–14.9)	2	12.5 (0.0–31.3)	11	9.3 (4.2–15.3)
2.00	31	23.1 (15.7–29.9)	5	31.3 (12.5–56.3)	26	22.0 (14.4–29.7)
3.00	35	26.1 (18.7–34.3)	0	0.0 (0.0–0.0)	35	29.7 (21.2–38.1)
4.00	36	26.9 (20.1–34.3)	6	37.5 (12.5–62.5)	30	25.4 (17.8–33.1)
5 and Above	19	14.2 (9.0–20.1)	3	18.8 (0.0–43.6)	16	13.6 (7.6–19.5)

### Association between lifetime use of drugs and ACEs

The ACEs were analysed against the lifetime use of substances so as to determine any association controlling for gender, educational level, employment and marital status, family history of substance abuse and family history of mental illness (Table 5).

**Table 5:** Prevalence and Adjusted Odds Ratio ^a^of Lifetime Use of Drugs by Category of ACE

Type of ACE		N	TOBACCOA.O.R	ALCOHOLA.O.R	CANNABISA.O.R	KHATA.O.R	SEDATIVESA.O.R
Physical abuse	No	103	1 (Ref.)	1 (Ref.)	1 (Ref.)	1 (Ref.)	1 (Ref.)
	Yes	31	1.4 (0.4–5.7)	5.6 (0.6–56.9)	2.5 (0.9–7.1)	1.2 (0.5–3.0)	**3.9 (1.4–11.2)**
Emotional abuse	No	104	1 (Ref.)	1 (Ref.)	1 (Ref.)	1 (Ref.)	1 (Ref.)
	Yes	30	**22.8 (1.6–332.9)**	5.0 (0.5–51.7)	1.9 (0.6–5.6)	1.0 (0.4–2.5)	*3.0 (1.0–9.1)* ^*b*^
Contact sexual abuse	No	99	1 (Ref.)	1 (Ref.)	1 (Ref.)	1 (Ref.)	1 (Ref.)
	Yes	35	1.1 (0.3–4.3)	1.2 (0.2–7.4)	1.1 (0.4–3.2)	0.7 (0.3–1.7)	1.7 (0.6–5.1)
Alcohol/drug user in household	No	76	1 (Ref.)	1 (Ref.)	1 (Ref.)	1 (Ref.)	1 (Ref.)
	Yes	58	1.8 (0.6–5.9)	1.3 (0.3–5.7)	0.8 (0.4–1.9)	2.0 (0.9–4.4)	0.7 (0.3–1.7)
Incarcerated household member	No	104	1 (Ref.)	1 (Ref.)	1 (Ref.)	1 (Ref.)	1 (Ref.)
	Yes	30	0.9 (0.2–4.5)	0.9 (0.1–5.6)	0.9 (0.3–2.6)	0.6 (0.2–1.8)	3.0 (0.9–10.0)
Someone with mental illness in household	No	113	1 (Ref.)	1 (Ref.)	1 (Ref.)	1 (Ref.)	1 (Ref.)
	Yes	21	UD	2.2 (0.2–23.5)	1.3 (0.4–4.1)	2.1 (0.7–6.6)	**3.0 (1.0–9.1)**
Household member treated violently	No	68	1 (Ref.)	1 (Ref.)	1 (Ref.)	1 (Ref.)	1 (Ref.)
	Yes	66	*3.6 (1.0–12.8)* ^*b*^	**14.6 (1.5–140.8)**	0.6 (0.3–1.4)	0.8 (0.4–1.8)	2.0 (0.8–5.2)
One or no parent	No	67	1 (Ref.)	1 (Ref.)	1 (Ref.)	1 (Ref.)	1 (Ref.)
	Yes	67	0.7 (0.2–2.1)	1.2 (0.3–5.1)	0.5 (0.2–1.1)	**0.4 (0.2–0.9)**	1.9 (0.7–5.2)
Emotional neglect	No	81	1 (Ref.)	1 (Ref.)	1 (Ref.)	1 (Ref.)	1 (Ref.)
	Yes	53	1.0 (0.3–3.4)	3.0 (0.5–16.6)	1.6 (0.7–3.8)	1.1 (0.5–2.5)	1.9 (0.7–4.7)
Physical neglect	No	118	1 (Ref.)	1 (Ref.)	1 (Ref.)	1 (Ref.)	1 (Ref.)
	Yes	16	UD	0.2 (0.0–1.1)	0.6 (0.2–2.3)	*0.3 (0.1–1.0)* ^*b*^	2.1 (0.6–8.4)
Bullying	No	117	1 (Ref.)	1 (Ref.)	1 (Ref.)	1 (Ref.)	1 (Ref.)
	Yes	17	UD	1.7 (0.2–17.3)	0.9 (0.3–3.0)	0.9 (0.3–2.8)	0.3 (0.1–2.0)
Community violence	No	93	1 (Ref.)	1 (Ref.)	1 (Ref.)	1 (Ref.)	1 (Ref.)
	Yes	41	4.2 (0.8–22.3)	7.0 (0.7–67.8)	1.7 (0.7–4.5)	**0.3 (0.1–0.8)**	1.3 (0.5–3.5)
Collective violence	No	79	1 (Ref.)	1 (Ref.)	1 (Ref.)	1 (Ref.)	1 (Ref.)
	Yes	55	1.1 (0.4–3.5)	1.6 (0.4–6.9)	0.8 (0.4–1.9)	**0.4 (0.2–0.9)**	2.5 (1.0–6.3)

^a^Adjusted For Gender, Education Level, Employment Status, Marital Status, Family History of Substance Abuse and Mental Illness; UD-Undetermined; Ref.-Referent category

^b^The values are more than 1.0, only rounded off to one decimal place for uniformity

Emotional abuse was found to be a significant predictor of using tobacco [A.O.*R* = 22.8 (1.6–332.9)]. Having a household member treated violently increased the risk of lifetime alcohol use [A.O.*R* = 14.6 (1.5–140.8)]. Lifetime sedative use was found to be positively associated with physical abuse, emotional abuse and having someone with mental illness in the household.

Having one or no parent, physical neglect, violence were all found to be protective against lifetime use of khat.

### Association between current use of drugs and ACEs

The ACEs were also analysed against the currently used substances so as to determine any association controlling for gender, educational level, employment and marital status, family history of substance abuse and family history of mental illness as depicted in Table 6.

**Table 6:** Prevalence and Adjusted Odds Ratio ^a^ Of Current Use of Drugs by Category of ACE

Type of ACE	Category	n (%)	Tobacco	Alcohol	Cannabis	Sedatives	Khat
			A.O.R	A.O.R	A.O.R	A.O.R	A.O.R
Physical abuse	No	103 (76.9)	1 (Ref)	1 (Ref)	1 (Ref)	1 (Ref)	1 (Ref)
	Yes	31 (23.1)	1.0 (0.3–2.9)	1.1 (0.4–3.4)	*2.9 (1.0–7.9)* ^*b*^	2.6 (0.8–8.4)	0.6 (0.2–1.7)
Emotional abuse	No	104 (77.6)	1 (Ref)	1 (Ref)	1 (Ref)	1 (Ref)	1 (Ref)
	Yes	30 (22.4)	**5.3 (1.2–23.9)**	0.8 (0.3–2.5)	2.5 (0.9–7.0)	**4.1 (1.2–14.2)**	0.5 (0.2–1.5)
Contact sexual abuse	No	99 (73.9)	1 (Ref)	1 (Ref)	1 (Ref)	1 (Ref)	1 (Ref)
	Yes	35 (26.1)	0.7 (0.2–2.0)	0.6 (0.2–1.8)	1.8 (0.7–5.0)	1.6 (0.5–5.5)	1.0 (0.4–2.5)
Alcohol/drug user in household	No	76 (56.7)	1 (Ref)	1 (Ref)	1 (Ref)	1 (Ref)	1 (Ref)
	Yes	58 (43.3)	1.1 (0.4–2.7)	0.8 (0.3–2.1)	1.2 (0.6–2.7)	0.8 (0.3–2.2)	2.1 (0.9–4.9)
Incarcerated household member	No	104 (77.6)	1 (Ref)	1 (Ref)	1 (Ref)	1 (Ref)	1 (Ref)
	Yes	30 (22.4)	1.5 (0.4–5.7)	0.5 (0.2–1.7)	1.1 (0.4–3.2)	2.3 (0.5–10.2)	0.9 (0.3–2.6)
Someone with mental illness in household	No	113 (84.3)	1 (Ref)	1 (Ref)	1 (Ref)	1 (Ref)	1 (Ref)
	Yes	21 (15.7)	*5.5 (1.0–28.8)* ^*b*^	0.5 (0.2–1.6)	0.9 (0.3–2.5)	1.6 (0.5–5.8)	2.4 (0.8–7.5)
Household member treated violently	No	68 (50.7)	1 (Ref)	1 (Ref)	1 (Ref)	1 (Ref)	1 (Ref)
	Yes	66 (49.3)	2.1 (0.8–5.5)	2.2 (0.8–6.2)	0.7 (0.3–1.6)	1.7 (0.6–5.2)	0.6 (0.3–1.3)
One or no parent	No	67 (50.0)	1 (Ref)	1 (Ref)	1 (Ref)	1 (Ref)	1 (Ref)
	Yes	67 (50.0)	1.0 (0.4–2.5)	0.9 (0.3–2.4)	0.5 (0.2–1.0)	2.2 (0.7–6.7)	**0.3 (0.1–0.8)**
Emotional neglect	No	81 (60.4)	1 (Ref)	1 (Ref)	1 (Ref)	1 (Ref)	1 (Ref)
	Yes	53 (39.6)	1.0 (0.4–2.6)	1.1 (0.4–2.9)	1.1 (0.5–2.5)	2.4 (0.8–7.1)	0.9 (0.4–2.0)
Physical neglect	No	118 (88.1)	1 (Ref)	1 (Ref)	1 (Ref)	1 (Ref)	1 (Ref)
	Yes	16(11.9)	5.7 (0.6–50.0)	0.4 (0.1–1.6)	0.9 (0.3–3.3)	*4.9 (1.0–23.2)* ^*b*^	0.5 (0.1–1.8)
Bullying	No	117 (87.3)	1 (Ref)	1 (Ref)	1 (Ref)	1 (Ref)	1 (Ref)
	Yes	17 (12.7)	1.8 (0.4–7.8)	0.4 (0.1–1.6)	0.7 (0.2–2.2)	0.0 (0.0-)	0.7 (0.2–2.4)
Community violence	No	93 (69.4)	1 (Ref)	1 (Ref)	1 (Ref)	1 (Ref)	1 (Ref)
	Yes	41 (30.6)	1.8 (0.6–5.4)	1.6 (0.5–5.0)	2.2 (0.9–5.5)	1.1 (0.3–3.5)	0.5 (0.2–1.2)
Collective violence	No	79 (59.0)	1 (Ref)	1 (Ref)	1 (Ref)	1 (Ref)	1 (Ref)
	Yes	55 (41.0)	1.1 (0.4–2.7)	2.4 (0.8–6.9)	1.2 (0.5–2.6)	1.7 (0.6–4.9)	0.5 (0.2–1.1)

^a^Adjusted For Gender, Education Level, Employment Status, Marital Status, Family History of Substance Abuse and Mental Illness; UD-Undetermined; Ref.-Referent category

^b^The values are more than 1.0, only that it was rounded off to one decimal place

Cannabis, tobacco and sedatives are the only substances which had significant positive correlation with experience of ACEs among the respondents. Having one or no parent was found to be protective against current use of khat.

## Discussion

The majority of patients being treated for substance use disorders in the current study were males (88.1%) which compares to previous studies [9, 29]. This could be due to the cultural attitudes and negative stigma attached to females who use substances hence, females avoid reporting about substance use or seeking treatment. Half (50%) of the respondents first used drugs when they were between the ages of 16 and 20. This is not surprising as substance use usually starts in adolescence due to peer pressure and curiosity leading to experimentation.

Alcohol, tobacco, cannabis and Khat were the most commonly used substances. Although, lifetime alcohol use was significantly associated with having been exposed to household member treated violently in childhood, there was no association found between current problematic use of alcohol and ACEs. Experiencing emotional abuse was found to be a significant predictor of problematic lifetime and/or current use of tobacco and sedatives. Surprisingly, experience of having one or no parent was found to be protective against current use of khat in this study. Sedative use was increased among participants who had experienced five or more ACEs.

Regarding the participant current use of substances; The most commonly used substances were alcohol (82.1%), tobacco (74.6%), cannabis (56.7%) and khat (46.3%) as has been demonstrated in earlier studies conducted in similar populations [10, 30]. Studies conducted in Kenya rural and urban health centres and Bugando hospital in Tanzania also found alcohol, tobacco, khat and cannabis to be the most frequently used substances [10, 30]. The substances most frequently used by respondents in the three studies are readily available and cost less compared to others such as cocaine. This could explain why most participants preferred them over others. The current findings however differ with a study conducted by Ndetei at the same facility. In their study, the most common substances reported by participants were opioids, sedatives and stimulants [9]. The reason for the variance could be because currently MNTRH offers outpatient treatment for opioid use disorders therefore few patients with opioid use disorders are treated as in- patients.

Most of the participants in this study used more than one substance with less than a tenth (9.7%) reporting use of only one substance. This agrees with the findings by a study in India where 91.9% of the participants reported to have poly-substance use.

Majority (92.5%) of the participants reported experiencing at least one ACE. This compares to findings from a study conducted in Saudi Arabia, where 18% of the participants reported no ACE exposure [31]. However, these rates are much higher than those from the ACE study in which 52% of the participants had experienced one or more ACEs [5]. The difference in the rates may be accounted for by the use of different questionnaires thereby difference in adversities screened for. The present study used the WHO Adverse childhood experiences international questionnaire whereas the ACE study used the ACE Study questionnaire which was constructed with questions from previously published surveys**.** Differences in prevalence estimates may also reflect variances brought about by different study settings i.e. Africa and non-African countries. Social factors will have a bearing on the occurrence of adverse childhood experiences and cultural expectations may determine which events individuals would consider as being adverse. The World Mental Health study, found that only 38.8% of the participants reported having experienced any ACE [32]. The lower prevalence could be explained by the different study populations; hospital based in the current study versus general population in the World Mental Health Study. Inpatients with substance use disorders are more likely to have experienced ACEs compared to the general population [11, 12].

Of those who reported experiencing any ACE majority (78.3%) reported more than one ACE. These results are comparable to previous studies [5, 31]. In the ACE study by Felitti and colleagues, 16% reported experiencing four or more ACEs [5]. Almuneef found that 32% of respondents reported four or more ACEs [31]. The reason for this could be that exposure to one ACE has been found to increase the likelihood of experiencing other childhood adversities [13, 33], this is also reflected in the current study.

The most commonly reported ACE was one or no parent which was reported by half (50%) of the respondents followed by household member treated violently (49%). This is different from the findings in the original ACE study where the commonest ACEs reported were physical abuse and substance abuse by household member (both reported by 28%) followed by sexual abuse (reported by 21%). The major differences in occurrence of ACE were those ACEs related to violence (community/collective violence and household member treated violently). The reason for this could be that the Felitti study did not assess for community or collective violence [5].

There were varied prevalence of ACEs by gender, however, in general males reported more ACEs as compared to females. This is also similar to findings in other ACE studies [5, 31]. The reasons for this could be that the number of females in the study was less than that of males. The other reason could be because most cultures are girl-sensitive; during childhood females tend to be protected from violent occurrences in the community than male children. Conversely, females could be reluctant to report adversity due to cultural reasons. Among the abuse ACEs males reported more physical and emotional abuse compared to females, while females reported more of contact sexual abuse.

Although there was no association between current alcohol use and ACEs, lifetime alcohol use was significantly associated with having been exposed to household member treated violently. This finding compares with results reported in earlier studies where the prevalence of alcoholism was higher among patients who reported parental alcohol use [33]. Other studies have also shown alcohol use to be associated with physical and sexual abuse and community violence [11, 14, 18].

Childhood exposure to emotional abuse increased the risk for lifetime tobacco use and current tobacco use by 22.8 and 5.3 times respectively. Tobacco use was also significantly associated with individuals who had been exposed to household member treated violently where they were about 4 times more likely to smoke tobacco in their lifetime. Having someone with mental illness in the household, increased risk of current tobacco use by five times. This is similar to findings by Anda and colleagues who reported an association between ACEs and early initiation of smoking [34]. Finding from another study stated that exposure to ACEs increased risk of continued smoking and heavy smoking in adulthood [35]. The mood elevating properties of nicotine may also explain why participants smoked to cope with emotional abuse and caregiver burden which may be a consequence of taking care of the mentally ill patient in the household.

Physical abuse positively predicted current use of cannabis which is comparable to previous studies [36, 37]. In a cohort study conducted by Alemu and colleagues, exposure to physical abuse was found to predict cannabis dependence (AOR = 2.81). Other experiences associated with cannabis dependence were emotional abuse and neglect [37]. In another study exposure to childhood sexual abuse but not childhood physical abuse was found to increase risk for cannabis abuse or dependence. The authors reported this as an unexpected finding [36]. The difference in the methodologies between the two studies may have contributed to this variation in findings.

Unexpectedly, contrary to previous studies [38] being exposed to violence, physical neglect and having one or no parent were found to be protective against using lifetime and/or current use of Khat in the current study. A study conducted among Somali refugees found exposure to traumatic experiences to predict khat usage [38]. Somali refugees who used khat screened positive for post-traumatic stress disorder. The diagnosis could have been an effect modifier which explains the variance with the current study. The different study designs: case control study among Somali refugees and descriptive cross-sectional in our study may also explain the different findings. Factors such as resilience, societal norms and coping strategies determine how one responds to adversities. None of the compared studies measured these factors which might also explain the variance in the findings.

We found no significant differences between the number of ACEs experienced and tobacco, alcohol, cannabis and khat use. However those who had experienced 5 ACEs were 15 times more likely to use sedatives as compared to those who had not experienced any form of ACEs. This is not surprising as high scores of ACEs have been linked to increased utilization of psychotropic medications such as anxiolytics in earlier studies [39]. Exposure to moderate or severe ACEs has been associated with a more disturbed sleep amongst patients with insomnia [40] which may explain the increased usage of sedatives among the group.

## Limitations

Over-reporting or under-reporting cannot be totally ruled out as a result of the use of self-report questionnaires. The data collected was retrospective which may have led to recall bias. Convenience sampling may result in sampling error and limits the generalizability of the findings beyond the sample. Effect modifiers which could alter the association of ACEs and substance use such as resilience and comorbid psychiatric illness were not measured in the study. The study was hospital based hence data collected was on patients who were admitted leaving out other patients with substance use disorders who may not be admitted. Therefore these findings may not be representation of all patients with substance use disorders.

## Conclusions and recommendations

There is a high prevalence of adverse childhood experiences among patients on treatment for substance use disorders. Early screening and prevention of ACEs could be of importance to reduce their effect and help in management of patients.

Alcohol, tobacco, cannabis and khat are the most commonly used substances. This could be due to easy availability and less cost of the above mentioned substances compared to other substances of abuse. The findings are important in providing guidance on where to focus treatment and prevention strategies for substance use.

The most commonly reported ACE was one or no parent and household member treated violently. Exposure to household member treated violently and emotional abuse were significantly associated with use of substances. This could form a basis to more focused studies to establish the relationship of the above ACEs and substance use disorders.

Further to the findings of this study, we recommend further studies on ACEs in different regions of the country in different settings (community and clinical) as well as comparison of ACE prevalence in general population and those with adverse health outcomes such as substance use disorders. The findings may be used to inform policies on prevention and management of substance use disorders.

The findings of this study highlight the need to address the high prevalence and consequences of childhood traumatic stressors.

## Abbreviations

ACE-IQ: Adverse Childhood experiences-International Questionnaire
ACEs: Adverse childhood experiences
ASSIST: Alcohol and substance involvement screening tool
CDC: Centers for disease control and prevention
DALY: Disability adjusted life years
MNTRH: Mathari national teaching and referral hospital
WHO: World health organization
